# Crystal structure of (*tert*-butyl­dimethyl­sil­yl)tri­phenyl­germane, Ph_3_Ge-SiMe_2_(*t*-Bu)

**DOI:** 10.1107/S2056989015022872

**Published:** 2015-12-06

**Authors:** Kirill V. Zaitsev, Galina S. Zaitseva, Sergey S. Karlov, Alexander A. Korlyukov

**Affiliations:** a Department of Chemistry, M.V. Lomonosov Moscow State University, Leninskie Gory 1/3, 119991 Moscow, Russian Federation; b A.N. Nesmeyanov Institute of Organoelement Compounds, Russian Academy of Sciences, Vavilova St. 28, 119991 Moscow, Russian Federation

**Keywords:** catenated compounds, silagermanes, C—H⋯π inter­actions, 6PE inter­actions, crystal structure

## Abstract

In the title compound, Ph_3_Ge-SiMe_2_(*t*-Bu) or C_24_H_30_GeSi, the Si and Ge atoms both possess a tetra­hedral coordination environment with C—*E*—C (*E* = Si, Ge) angles in the range 104.47 (5)–114.67 (5)°. The mol­ecule adopts an eclipsed conformation, with three torsion angles less than 29.5°. In the crystal, neighbouring mol­ecules are combined to dimers by six T-shaped C—H⋯π inter­actions, forming sixfold phenyl embraces (6PE).

## Related literature   

For general background to the chemistry of Group 14 element catenated compounds, see: Marschner & Hlina (2013 ▸); Amadoruge & Weinert (2008 ▸); Párkányi *et al.* (1986 ▸); Leigh *et al.* (1997 ▸). As apart of our studies of the chemistry of oligogermanium compounds (Zaitsev *et al.* 2012 ▸, 2013 ▸, 2014*a* ▸,*b* ▸), the title compound was obtained and studied. For related crystal structures of silagermanes, see: Zaitsev *et al.* (2015 ▸). The 6PE inter­actions are intensively discussed in Scudder & Dance (2000 ▸); Steiner (2000 ▸); Churakov *et al.* (2005 ▸).
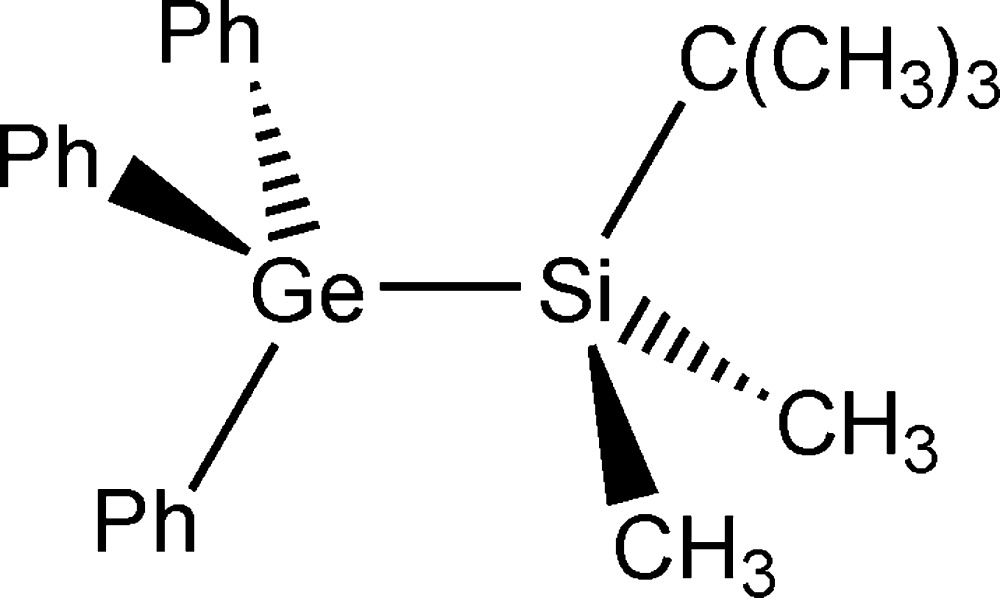


## Experimental   

### Crystal data  

C_24_H_30_GeSi
*M* *_r_* = 419.16
Monoclinic, 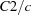

*a* = 13.5332 (6) Å
*b* = 14.9825 (7) Å
*c* = 22.7179 (13) Å
β = 106.2048 (10)°
*V* = 4423.3 (4) Å^3^
*Z* = 8
Mo *K*α radiation
μ = 1.44 mm^−1^
*T* = 120 K
0.32 × 0.29 × 0.24 mm

### Data collection  

Bruker SMART APEXII CCD area-detector diffractometer
Absorption correction: multi-scan (*SADABS*; Bruker, 2013 ▸) *T*_min_ = 0.720, *T*_max_ = 0.862
32242 measured reflections
7990 independent reflections
6137 reflections with *I* > 2σ(*I*)
*R*_int_ = 0.043

### Refinement  

*R*[*F* ^2^ > 2σ(*F* ^2^)] = 0.033
*wR*(*F* ^2^) = 0.071
*S* = 1.01
7990 reflections
240 parameters
H-atom parameters constrained
Δρ_max_ = 0.43 e Å^−3^
Δρ_min_ = −0.37 e Å^−3^

### 

Data collection: *APEX2* (Bruker, 2013 ▸); cell refinement: *SAINT* (Bruker, 2013 ▸); data reduction: *SAINT*; program(s) used to solve structure: *SHELXS2014* (Sheldrick, 2008 ▸); program(s) used to refine structure: *SHELXL2014* (Sheldrick, 2015 ▸); molecular graphics: *OLEX2* (Dolomanov *et al.*, 2009 ▸); software used to prepare material for publication: *OLEX2*.

## Supplementary Material

Crystal structure: contains datablock(s) I. DOI: 10.1107/S2056989015022872/im2474sup1.cif

Structure factors: contains datablock(s) I. DOI: 10.1107/S2056989015022872/im2474Isup2.hkl

Supporting information file. DOI: 10.1107/S2056989015022872/im2474Isup3.mol

Supporting information file. DOI: 10.1107/S2056989015022872/im2474Isup4.cml

**Fig. 1.**
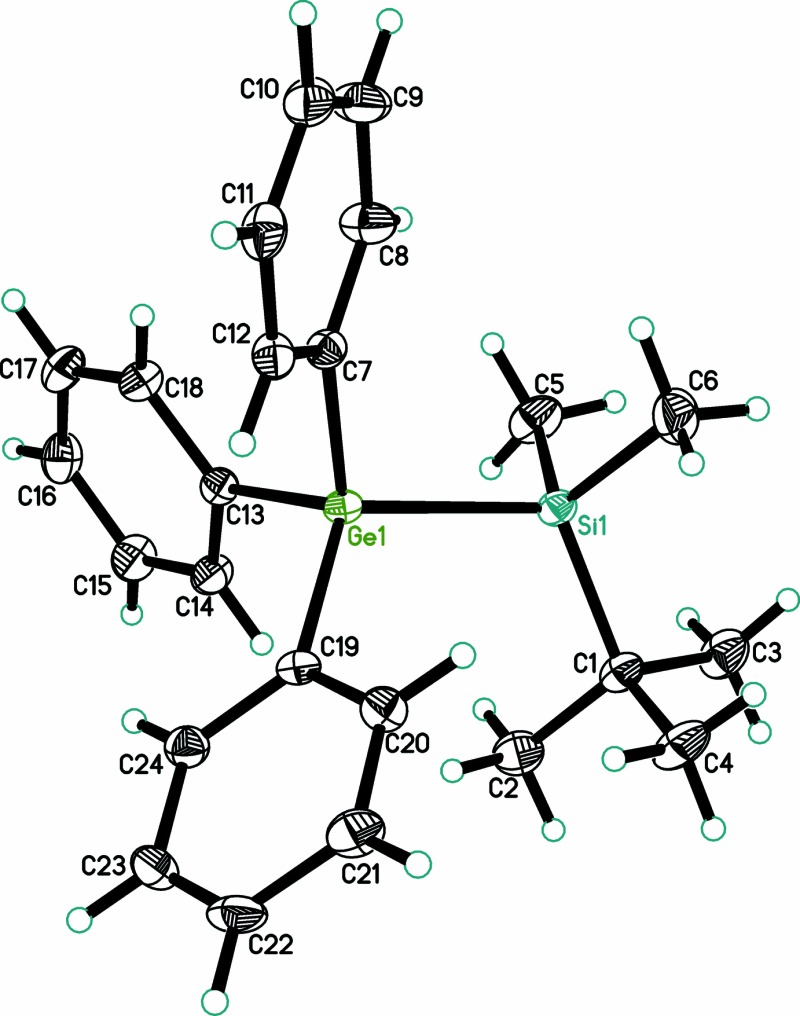
Molecular structure of the title compound, with displacement ellipsoids shown at the 50% probability level.

. DOI: 10.1107/S2056989015022872/im2474fig1.tif
Mol­ecular structure of the title compound, with displacement ellipsoids shown at the 50% probability level.

**Fig. 2.**
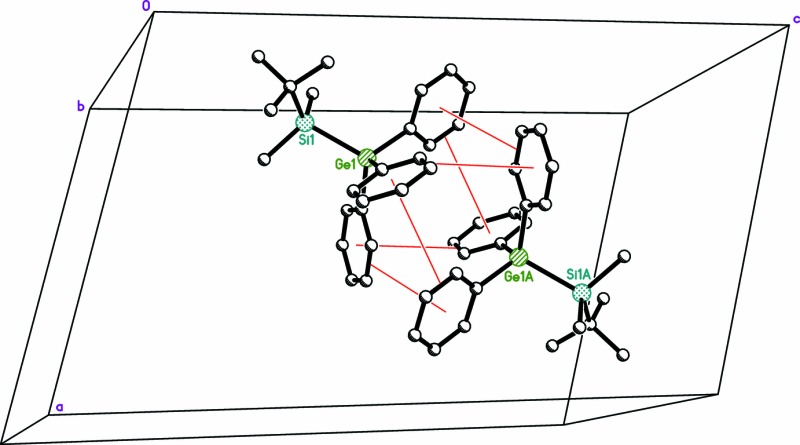
Dimers formed by 6PE interactions between adjacent molecules.

. DOI: 10.1107/S2056989015022872/im2474fig2.tif
Dimers formed by 6PE inter­actions between adjacent mol­ecules.

CCDC reference: 1439529

Additional supporting information:  crystallographic information; 3D view; checkCIF report

## supplementary crystallographic information

### S1. Structural commentary

In the title compound, Ph_3_Ge-SiMe_2_(*t*-Bu), both Si and Ge atoms possess tetra­hedral coordination environments with C—E—C angles ranging within 104.47 (5)- 114.67 (5) °. The Ge—Si bond length (2.4026 (4) Å) is slightly longer than in the closely related compound Ph_3_Ge-SiMe_3_ (2.384 (1) Å (Párkányi *et al.*, 1986). The molecule adopts an eclipsed conformation with three torsion angles less than 29.5°.

In the crystal, neighbouring molecules are combined to dimers by six T-shaped C—H···π inter­actions forming sixfold phenyl embraces (6PE, Steiner, 2000; Churakov *et al.*, 2005). As expected for 6PE-bonded molecules, the C_ax_—Ge···Ge angle is almost linear − 175.9° (Fig. 2; Scudder & Dance, 2000).

The title compound is isostructural with the corresponding silicon complex Ph_3_Si-SiMe_2_(*t*-Bu) (Leigh *et al.*, 1997).

### S2. Synthesis and crystallization

The synthetic procedure leading to the title compound was reported by us earlier (Zaitsev *et al.*, 2014*b*) to give a white crystalline material in good yield (86%) by the reaction of Ph_3_GeLi (generated *in situ* from equimolar amounts of Ph_3_GeH and *n*-BuLi at room temperature in Et_2_O) with *t*-BuMe_2_SiCl in di­ethyl ether. Solvent-free crystals of the title compound suitable for X-Ray analysis were obtained after recrystallization from *n*-hexane at room temperature.

### S3. Refinement

Crystal data, data collection and structure refinement details are summarized in Table 1. A l l non-hydrogen atoms were refined with anisotropic thermal parameters.

All hydrogen atoms were placed in calculated positions and refined using a riding model, with C—H = 0.93–0.96 Å, and with *U*_iso_(H) = 1.2 *U*_eq_(C) for aromatic H atoms or 1.5 *U*_eq_(C) for methyl H atoms. A rotating model was applied to the methyl groups.

### Crystal data

**Table:**

C_24_H_30_GeSi	*F*(000) = 1760
*M**_r_* = 419.16	*D*_x_ = 1.259 Mg m^−^^3^
Monoclinic, *C*2/*c*	Mo *K*α radiation, λ = 0.71073 Å
*a* = 13.5332 (6) Å	Cell parameters from 8565 reflections
*b* = 14.9825 (7) Å	θ = 2.5–31.7°
*c* = 22.7179 (13) Å	µ = 1.44 mm^−^^1^
β = 106.2048 (10)°	*T* = 120 K
*V* = 4423.3 (4) Å^3^	Irregular, colourless
*Z* = 8	0.32 × 0.29 × 0.24 mm

### Data collection

**Table:**

Bruker SMART APEXII CCD area-detector diffractometer	7990 independent reflections
Radiation source: sealed tube	6137 reflections with *I* > 2σ(*I*)
Graphite monochromator	*R*_int_ = 0.043
Detector resolution: 8 pixels mm^-1^	θ_max_ = 32.6°, θ_min_ = 1.9°
ω and φ scans	*h* = −19→20
Absorption correction: multi-scan (*SADABS*; Bruker, 2013)	*k* = −22→22
*T*_min_ = 0.720, *T*_max_ = 0.862	*l* = −33→34
32242 measured reflections	

### Refinement

**Table:**

Refinement on *F*^2^	0 restraints
Least-squares matrix: full	Hydrogen site location: inferred from neighbouring sites
*R*[*F*^2^ > 2σ(*F*^2^)] = 0.033	H-atom parameters constrained
*wR*(*F*^2^) = 0.071	*w* = 1/[σ^2^(*F*_o_^2^) + (0.0303*P*)^2^ + 1.8636*P*] where *P* = (*F*_o_^2^ + 2*F*_c_^2^)/3
*S* = 1.01	(Δ/σ)_max_ = 0.002
7990 reflections	Δρ_max_ = 0.43 e Å^−^^3^
240 parameters	Δρ_min_ = −0.37 e Å^−^^3^

### Special details

**Table:**

Experimental. Absorption correctgion: *SADABS2008*/1 (Bruker,2008) was used for absorption correction. wR2(int) was 0.0820 before and 0.0431 after correction. The Ratio of minimum to maximum transmission is 0.8344. The λ/2 correction factor is 0.0015.
Geometry. All e.s.d.'s (except the e.s.d. in the dihedral angle between two l.s. planes) are estimated using the full covariance matrix. The cell e.s.d.'s are taken into account individually in the estimation of e.s.d.'s in distances, angles and torsion angles; correlations between e.s.d.'s in cell parameters are only used when they are defined by crystal symmetry. An approximate (isotropic) treatment of cell e.s.d.'s is used for estimating e.s.d.'s involving l.s. planes.

### Fractional atomic coordinates and isotropic or equivalent isotropic displacement parameters (Å^2^)

**Table:**

	*x*	*y*	*z*	*U*_iso_*/*U*_eq_	
Ge1	0.36354 (2)	0.03558 (2)	0.37248 (2)	0.01369 (4)	
Si1	0.26294 (3)	0.07258 (3)	0.27044 (2)	0.01566 (8)	
C1	0.19468 (11)	−0.02626 (9)	0.22333 (7)	0.0193 (3)	
C2	0.14760 (13)	−0.08808 (11)	0.26222 (8)	0.0280 (3)	
H2A	0.2015	−0.1135	0.2947	0.042*	
H2B	0.1099	−0.1350	0.2369	0.042*	
H2C	0.1020	−0.0544	0.2794	0.042*	
C3	0.10842 (13)	0.00982 (12)	0.16951 (8)	0.0286 (4)	
H3A	0.0582	0.0399	0.1849	0.043*	
H3B	0.0763	−0.0388	0.1437	0.043*	
H3C	0.1368	0.0509	0.1462	0.043*	
C4	0.26903 (13)	−0.08070 (11)	0.19725 (8)	0.0293 (4)	
H4A	0.2967	−0.0432	0.1715	0.044*	
H4B	0.2327	−0.1298	0.1737	0.044*	
H4C	0.3241	−0.1032	0.2303	0.044*	
C5	0.16501 (12)	0.15374 (11)	0.28177 (8)	0.0288 (4)	
H5A	0.1152	0.1229	0.2971	0.043*	
H5B	0.1311	0.1814	0.2433	0.043*	
H5C	0.1984	0.1986	0.3107	0.043*	
C6	0.34687 (14)	0.13018 (13)	0.22957 (8)	0.0340 (4)	
H6A	0.3806	0.1798	0.2536	0.051*	
H6B	0.3056	0.1512	0.1905	0.051*	
H6C	0.3976	0.0891	0.2235	0.051*	
C7	0.48101 (10)	0.11767 (9)	0.39511 (6)	0.0160 (3)	
C8	0.46614 (12)	0.20997 (10)	0.38666 (7)	0.0244 (3)	
H8	0.3997	0.2322	0.3713	0.029*	
C9	0.54855 (13)	0.26859 (11)	0.40074 (8)	0.0287 (4)	
H9	0.5371	0.3295	0.3948	0.034*	
C10	0.64792 (12)	0.23684 (11)	0.42370 (7)	0.0258 (3)	
H10	0.7032	0.2763	0.4332	0.031*	
C11	0.66436 (11)	0.14632 (11)	0.43231 (7)	0.0223 (3)	
H11	0.7310	0.1247	0.4475	0.027*	
C12	0.58171 (11)	0.08718 (10)	0.41835 (6)	0.0185 (3)	
H12	0.5939	0.0264	0.4246	0.022*	
C13	0.28202 (11)	0.05273 (9)	0.43088 (6)	0.0159 (3)	
C14	0.19262 (11)	0.00334 (10)	0.42642 (7)	0.0210 (3)	
H14	0.1719	−0.0391	0.3956	0.025*	
C15	0.13399 (12)	0.01638 (11)	0.46710 (7)	0.0254 (3)	
H15	0.0750	−0.0174	0.4635	0.031*	
C16	0.16361 (12)	0.07993 (11)	0.51311 (7)	0.0252 (3)	
H16	0.1242	0.0891	0.5402	0.030*	
C17	0.25164 (12)	0.12936 (11)	0.51847 (7)	0.0251 (3)	
H17	0.2717	0.1719	0.5494	0.030*	
C18	0.31062 (11)	0.11602 (10)	0.47793 (7)	0.0203 (3)	
H18	0.3700	0.1497	0.4821	0.024*	
C19	0.41975 (10)	−0.08517 (9)	0.38053 (6)	0.0151 (3)	
C20	0.47948 (11)	−0.11181 (10)	0.34217 (7)	0.0192 (3)	
H20	0.4907	−0.0719	0.3134	0.023*	
C21	0.52196 (12)	−0.19630 (10)	0.34629 (7)	0.0238 (3)	
H21	0.5611	−0.2128	0.3203	0.029*	
C22	0.50633 (12)	−0.25642 (10)	0.38912 (7)	0.0250 (3)	
H22	0.5351	−0.3132	0.3921	0.030*	
C23	0.44760 (12)	−0.23154 (10)	0.42747 (7)	0.0247 (3)	
H23	0.4366	−0.2718	0.4561	0.030*	
C24	0.40487 (11)	−0.14636 (10)	0.42328 (7)	0.0194 (3)	
H24	0.3659	−0.1302	0.4494	0.023*	

### Atomic displacement parameters (Å^2^)

**Table:**

	*U*^11^	*U*^22^	*U*^33^	*U*^12^	*U*^13^	*U*^23^
Ge1	0.01210 (7)	0.01250 (7)	0.01693 (7)	−0.00005 (6)	0.00481 (5)	−0.00168 (6)
Si1	0.01585 (18)	0.01453 (18)	0.01666 (18)	0.00156 (14)	0.00463 (14)	−0.00171 (14)
C1	0.0175 (6)	0.0189 (7)	0.0196 (7)	0.0025 (5)	0.0021 (5)	−0.0043 (5)
C2	0.0260 (8)	0.0266 (8)	0.0293 (8)	−0.0083 (7)	0.0042 (7)	−0.0038 (7)
C3	0.0249 (8)	0.0306 (9)	0.0245 (8)	0.0037 (7)	−0.0025 (6)	−0.0045 (7)
C4	0.0281 (8)	0.0279 (8)	0.0297 (9)	0.0060 (7)	0.0044 (7)	−0.0127 (7)
C5	0.0269 (8)	0.0245 (8)	0.0309 (9)	0.0112 (7)	0.0013 (7)	−0.0060 (7)
C6	0.0410 (10)	0.0391 (10)	0.0243 (8)	−0.0134 (8)	0.0132 (7)	−0.0001 (7)
C7	0.0152 (6)	0.0170 (6)	0.0161 (6)	−0.0019 (5)	0.0050 (5)	−0.0015 (5)
C8	0.0198 (7)	0.0182 (7)	0.0322 (8)	−0.0008 (6)	0.0024 (6)	−0.0008 (6)
C9	0.0291 (8)	0.0182 (7)	0.0356 (9)	−0.0064 (6)	0.0039 (7)	−0.0003 (7)
C10	0.0231 (7)	0.0305 (8)	0.0225 (7)	−0.0123 (6)	0.0040 (6)	−0.0018 (6)
C11	0.0148 (6)	0.0328 (8)	0.0178 (7)	−0.0026 (6)	0.0021 (5)	0.0009 (6)
C12	0.0176 (7)	0.0208 (7)	0.0168 (6)	−0.0004 (5)	0.0044 (5)	0.0014 (5)
C13	0.0151 (6)	0.0158 (6)	0.0167 (6)	0.0016 (5)	0.0044 (5)	−0.0001 (5)
C14	0.0220 (7)	0.0226 (7)	0.0199 (7)	−0.0051 (6)	0.0082 (6)	−0.0044 (6)
C15	0.0220 (7)	0.0318 (9)	0.0251 (8)	−0.0066 (6)	0.0108 (6)	−0.0023 (6)
C16	0.0249 (8)	0.0334 (9)	0.0206 (7)	0.0041 (7)	0.0117 (6)	−0.0005 (6)
C17	0.0277 (8)	0.0277 (8)	0.0200 (7)	0.0009 (6)	0.0069 (6)	−0.0073 (6)
C18	0.0189 (7)	0.0209 (7)	0.0209 (7)	−0.0017 (6)	0.0051 (6)	−0.0037 (6)
C19	0.0135 (6)	0.0123 (6)	0.0189 (6)	−0.0003 (5)	0.0034 (5)	−0.0023 (5)
C20	0.0184 (7)	0.0187 (7)	0.0215 (7)	0.0019 (5)	0.0073 (5)	0.0006 (6)
C21	0.0216 (7)	0.0216 (7)	0.0290 (8)	0.0051 (6)	0.0083 (6)	−0.0045 (6)
C22	0.0256 (8)	0.0124 (6)	0.0327 (8)	0.0033 (6)	0.0010 (6)	−0.0025 (6)
C23	0.0306 (8)	0.0173 (7)	0.0239 (8)	−0.0016 (6)	0.0038 (6)	0.0047 (6)
C24	0.0201 (7)	0.0183 (7)	0.0201 (7)	−0.0017 (6)	0.0060 (5)	−0.0009 (6)

### Geometric parameters (Å, º)

**Table:**

Ge1—Si1	2.4026 (4)	C9—H9	0.9300
Ge1—C7	1.9618 (14)	C9—C10	1.384 (2)
Ge1—C13	1.9648 (14)	C10—H10	0.9300
Ge1—C19	1.9512 (13)	C10—C11	1.379 (2)
Si1—C1	1.9078 (15)	C11—H11	0.9300
Si1—C5	1.8687 (15)	C11—C12	1.393 (2)
Si1—C6	1.8670 (17)	C12—H12	0.9300
C1—C2	1.536 (2)	C13—C14	1.398 (2)
C1—C3	1.534 (2)	C13—C18	1.400 (2)
C1—C4	1.537 (2)	C14—H14	0.9300
C2—H2A	0.9600	C14—C15	1.389 (2)
C2—H2B	0.9600	C15—H15	0.9300
C2—H2C	0.9600	C15—C16	1.388 (2)
C3—H3A	0.9600	C16—H16	0.9300
C3—H3B	0.9600	C16—C17	1.379 (2)
C3—H3C	0.9600	C17—H17	0.9300
C4—H4A	0.9600	C17—C18	1.391 (2)
C4—H4B	0.9600	C18—H18	0.9300
C4—H4C	0.9600	C19—C20	1.4019 (19)
C5—H5A	0.9600	C19—C24	1.390 (2)
C5—H5B	0.9600	C20—H20	0.9300
C5—H5C	0.9600	C20—C21	1.383 (2)
C6—H6A	0.9600	C21—H21	0.9300
C6—H6B	0.9600	C21—C22	1.385 (2)
C6—H6C	0.9600	C22—H22	0.9300
C7—C8	1.403 (2)	C22—C23	1.384 (2)
C7—C12	1.3939 (19)	C23—H23	0.9300
C8—H8	0.9300	C23—C24	1.393 (2)
C8—C9	1.385 (2)	C24—H24	0.9300
			
C7—Ge1—Si1	107.93 (4)	C7—C8—H8	119.4
C7—Ge1—C13	107.92 (6)	C9—C8—C7	121.19 (15)
C13—Ge1—Si1	110.34 (4)	C9—C8—H8	119.4
C19—Ge1—Si1	113.92 (4)	C8—C9—H9	119.9
C19—Ge1—C7	106.89 (6)	C10—C9—C8	120.28 (15)
C19—Ge1—C13	109.61 (6)	C10—C9—H9	119.9
C1—Si1—Ge1	114.67 (5)	C9—C10—H10	120.2
C5—Si1—Ge1	104.47 (5)	C11—C10—C9	119.52 (14)
C5—Si1—C1	109.35 (7)	C11—C10—H10	120.2
C6—Si1—Ge1	109.08 (6)	C10—C11—H11	119.8
C6—Si1—C1	110.26 (7)	C10—C11—C12	120.39 (14)
C6—Si1—C5	108.72 (9)	C12—C11—H11	119.8
C2—C1—Si1	111.05 (10)	C7—C12—H12	119.5
C2—C1—C4	108.84 (13)	C11—C12—C7	121.06 (14)
C3—C1—Si1	108.38 (10)	C11—C12—H12	119.5
C3—C1—C2	109.00 (13)	C14—C13—Ge1	121.31 (10)
C3—C1—C4	108.31 (13)	C14—C13—C18	117.60 (13)
C4—C1—Si1	111.20 (10)	C18—C13—Ge1	121.09 (11)
C1—C2—H2A	109.5	C13—C14—H14	119.3
C1—C2—H2B	109.5	C15—C14—C13	121.38 (14)
C1—C2—H2C	109.5	C15—C14—H14	119.3
H2A—C2—H2B	109.5	C14—C15—H15	120.0
H2A—C2—H2C	109.5	C16—C15—C14	119.93 (15)
H2B—C2—H2C	109.5	C16—C15—H15	120.0
C1—C3—H3A	109.5	C15—C16—H16	120.1
C1—C3—H3B	109.5	C17—C16—C15	119.72 (14)
C1—C3—H3C	109.5	C17—C16—H16	120.1
H3A—C3—H3B	109.5	C16—C17—H17	119.8
H3A—C3—H3C	109.5	C16—C17—C18	120.36 (14)
H3B—C3—H3C	109.5	C18—C17—H17	119.8
C1—C4—H4A	109.5	C13—C18—H18	119.5
C1—C4—H4B	109.5	C17—C18—C13	121.01 (14)
C1—C4—H4C	109.5	C17—C18—H18	119.5
H4A—C4—H4B	109.5	C20—C19—Ge1	118.88 (10)
H4A—C4—H4C	109.5	C24—C19—Ge1	123.24 (10)
H4B—C4—H4C	109.5	C24—C19—C20	117.87 (13)
Si1—C5—H5A	109.5	C19—C20—H20	119.3
Si1—C5—H5B	109.5	C21—C20—C19	121.30 (14)
Si1—C5—H5C	109.5	C21—C20—H20	119.3
H5A—C5—H5B	109.5	C20—C21—H21	120.0
H5A—C5—H5C	109.5	C20—C21—C22	120.06 (14)
H5B—C5—H5C	109.5	C22—C21—H21	120.0
Si1—C6—H6A	109.5	C21—C22—H22	120.2
Si1—C6—H6B	109.5	C23—C22—C21	119.62 (14)
Si1—C6—H6C	109.5	C23—C22—H22	120.2
H6A—C6—H6B	109.5	C22—C23—H23	119.9
H6A—C6—H6C	109.5	C22—C23—C24	120.25 (14)
H6B—C6—H6C	109.5	C24—C23—H23	119.9
C8—C7—Ge1	120.48 (11)	C19—C24—C23	120.90 (14)
C12—C7—Ge1	121.93 (11)	C19—C24—H24	119.6
C12—C7—C8	117.57 (13)	C23—C24—H24	119.6
			
Ge1—C7—C8—C9	−178.11 (13)	C13—C14—C15—C16	0.4 (2)
Ge1—C7—C12—C11	177.92 (11)	C14—C13—C18—C17	−0.3 (2)
Ge1—C13—C14—C15	−178.96 (12)	C14—C15—C16—C17	−0.5 (3)
Ge1—C13—C18—C17	178.66 (12)	C15—C16—C17—C18	0.2 (2)
Ge1—C19—C20—C21	179.40 (11)	C16—C17—C18—C13	0.2 (2)
Ge1—C19—C24—C23	−179.38 (11)	C18—C13—C14—C15	0.0 (2)
C7—C8—C9—C10	−0.1 (3)	C19—C20—C21—C22	−0.3 (2)
C8—C7—C12—C11	−0.3 (2)	C20—C19—C24—C23	−0.4 (2)
C8—C9—C10—C11	0.2 (2)	C20—C21—C22—C23	0.3 (2)
C9—C10—C11—C12	−0.3 (2)	C21—C22—C23—C24	−0.3 (2)
C10—C11—C12—C7	0.4 (2)	C22—C23—C24—C19	0.4 (2)
C12—C7—C8—C9	0.2 (2)	C24—C19—C20—C21	0.3 (2)
