# Supplementary figures and images for: Crystal structure of (tert-butyl­dimethyl­sil­yl)tri­phenyl­germane, Ph3Ge-SiMe2(t-Bu)
Source: Acta Crystallogr E Crystallogr Commun. 2015 Dec 6;71(Pt 12):o1015–6. doi: 10.1107/S2056989015022872 (PMC4719953; doi:10.1107/S2056989015022872)

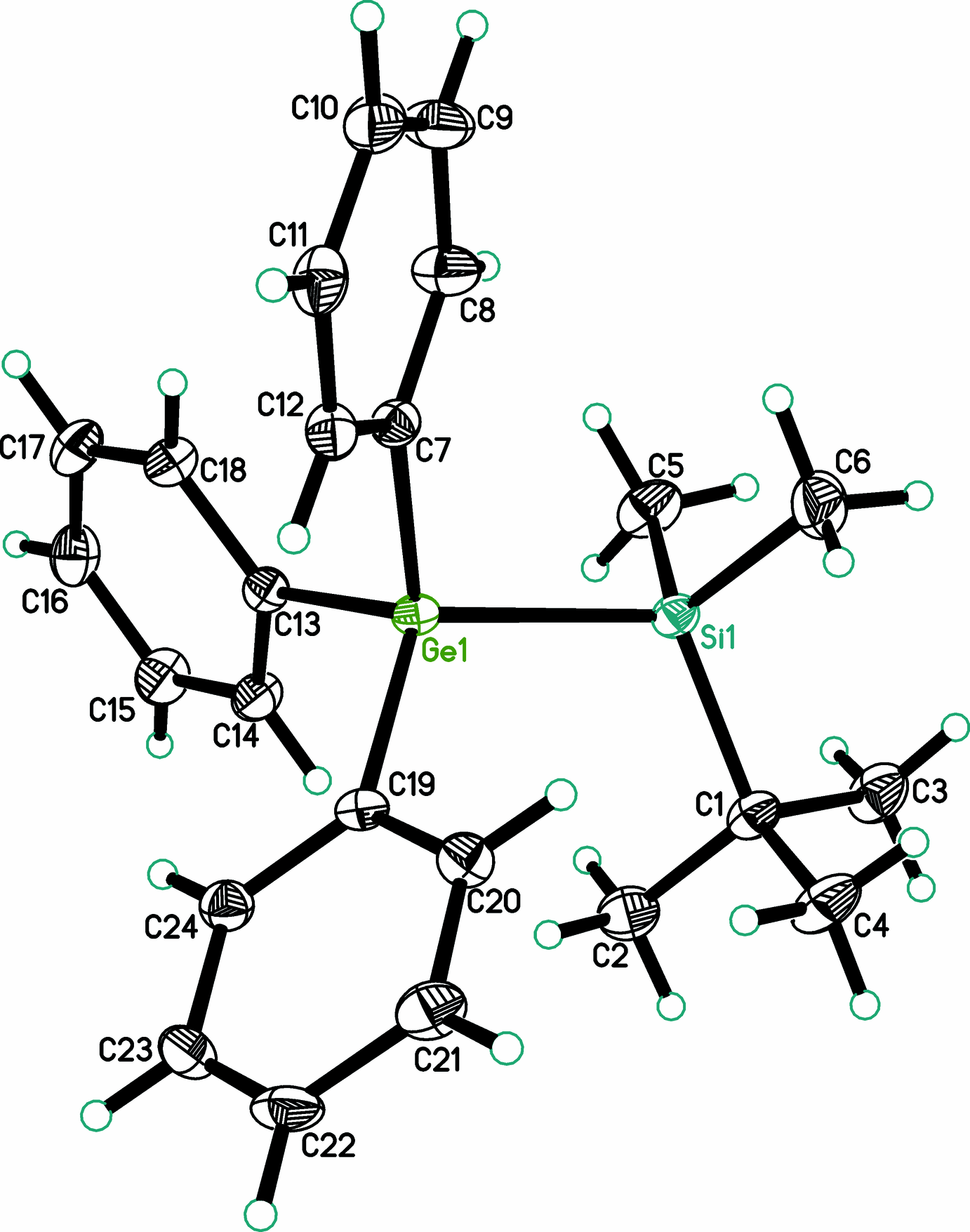

Supplement: Supplementary file 5 [file e-71-o1015-fig1.tif]

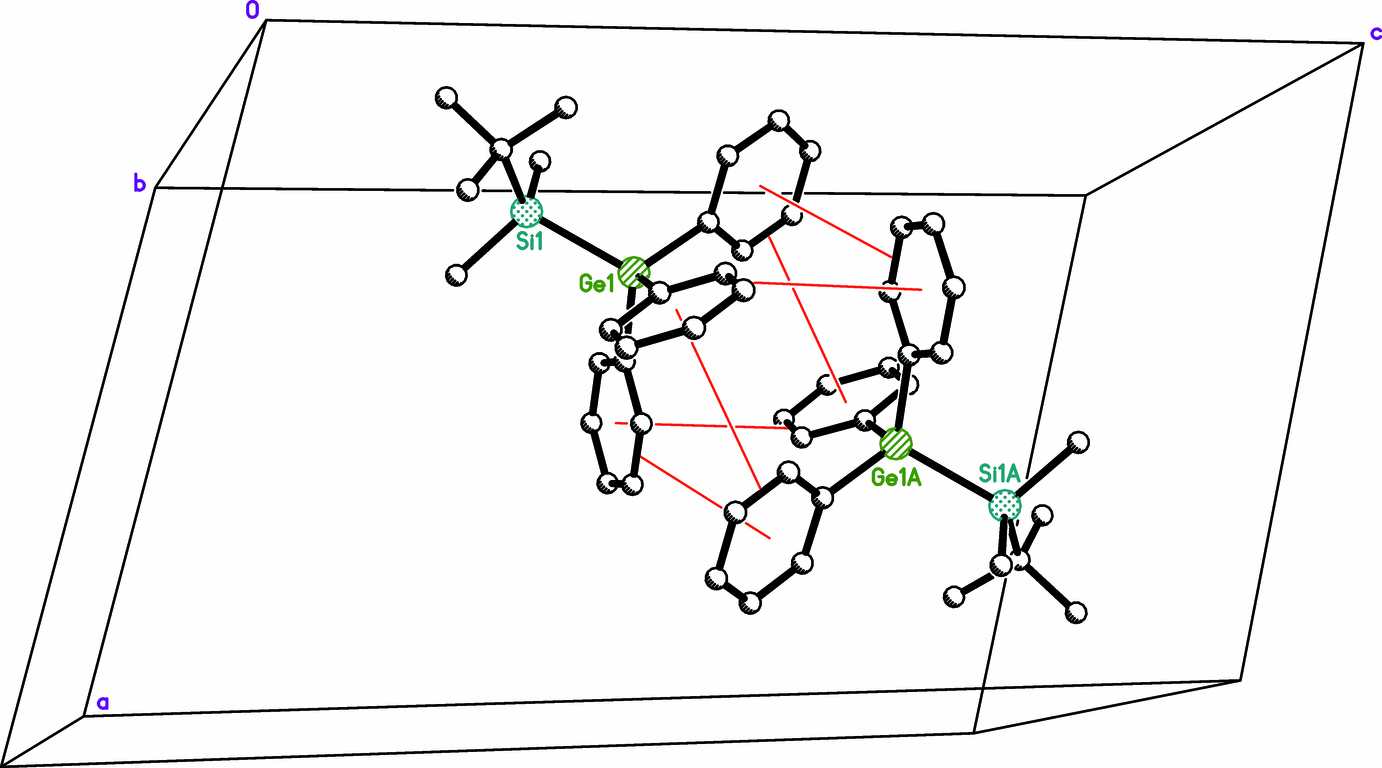

Supplement: Supplementary file 6 [file e-71-o1015-fig2.tif]
